# The non-deterministic genotype–phenotype map of RNA secondary structure

**DOI:** 10.1098/rsif.2023.0132

**Published:** 2023-08-23

**Authors:** Paula García-Galindo, Sebastian E. Ahnert, Nora S. Martin

**Affiliations:** ^1^ Department of Chemical Engineering and Biotechnology, University of Cambridge, Philippa Fawcett Drive, Cambridge CB3 0AS, UK; ^2^ The Alan Turing Institute, 96 Euston Road, London NW1 2DB, UK; ^3^ Rudolf Peierls Centre for Theoretical Physics, Beecroft Building, Parks Road, Oxford OX1 3PU, UK

**Keywords:** RNA, robustness, evolvability, many-to-many genotype–phenotype map

## Abstract

Selection and variation are both key aspects in the evolutionary process. Previous research on the mapping between molecular sequence (genotype) and molecular fold (phenotype) has shown the presence of several structural properties in different biological contexts, implying that these might be universal in evolutionary spaces. The deterministic genotype–phenotype (GP) map that links short RNA sequences to minimum free energy secondary structures has been studied extensively because of its computational tractability and biologically realistic nature. However, this mapping ignores the phenotypic plasticity of RNA. We define a GP map that incorporates non-deterministic (ND) phenotypes, and take RNA as a case study; we use the Boltzmann probability distribution of folded structures and examine the structural properties of ND GP maps for RNA sequences of length 12 and coarse-grained RNA structures of length 30 (RNAshapes30). A framework is presented to study robustness, evolvability and neutral spaces in the ND map. This framework is validated by demonstrating close correspondence between the ND quantities and sample averages of their deterministic counterparts. When using the ND framework we observe the same structural properties as in the deterministic GP map, such as bias, negative correlation between genotypic robustness and evolvability, and positive correlation between phenotypic robustness and evolvability.

## 1. Introduction

Biological information is stored in our genome in DNA and RNA as linear sequences composed of a nucleotide quaternary alphabet [1]. In nature, RNA is commonly found as a single-stranded sequence that can fold, through nucleotide interactions, into a three-dimensional molecular structure. For short RNAs, this three-dimensional structure can be coarse-grained to a two-dimensional representation of the fold (secondary structure) [2]. The biological functions that RNA executes in the cell depend on its folded structure [2], and so a direct link between RNA sequence, its minimum free energy (MFE) fold, and its function can be constructed [3,4]. The sequence-structure mapping can be called the genotype–phenotype (GP) map for RNA, since the sequence is the biological information carrier (genotype) and the fold has a direct implication on function and therefore selection (phenotype) [4].

For RNA of length *L*, the GP map involves all possible *L*-sequences mapped to their respective MFE secondary structures, in which each sequence is connected to its point-mutation neighbours [5]. The GP map can then be interpreted as a road-map outlining the accessibility of phenotypes in terms of point mutations of genotypes [5]. Constructing this mapping is tractable for short RNAs with the currently available computational tools [6,7], which is one of the main reasons why the GP map of RNA secondary structure has been studied so extensively [5].

The study of different GP maps which are not necessarily sequence-structure but represent a different phenotype (e.g. for proteins [8–10], metabolic networks [5] and gene regulatory networks (GRNs) [11]) has shown the prevalence of structural properties of the GP map that are common with the RNA sequence-structure map, suggesting that the GP maps exhibit properties that are characteristic of evolutionary spaces in general [1,5]. These properties include redundancy, bias, correlations between evolvability (i.e. the potential for phenotypic changes through mutations) and robustness (i.e. the probability of phenotype-preserving mutations), and the presence of neutral spaces and genetic correlations [5]. First, most GP maps display redundancy, which means that there are many genotypes mapping to a single phenotype, for example many sequences folding into a single structure in the RNA GP map. The set of genotypes mapping to the same phenotype is called a neutral space [1], or a neutral network if they are connected by point mutations [1,12]. Secondly, most GP maps are biased, which means that some phenotypes are much more redundant than others and thus have much larger neutral spaces. Thirdly, GP maps show a correlation between evolvability and robustness that is negative at genotypic level and positive at the phenotypic level [13]. Finally, GP maps usually display genetic correlations, which means that robustness is much higher than we would expect in a null model that simply accounts for neutral space sizes [14]. It is neutral spaces and networks in GP maps that explain the emergence of the more general concept of biological robustness [1,13].

**Figure 1.**
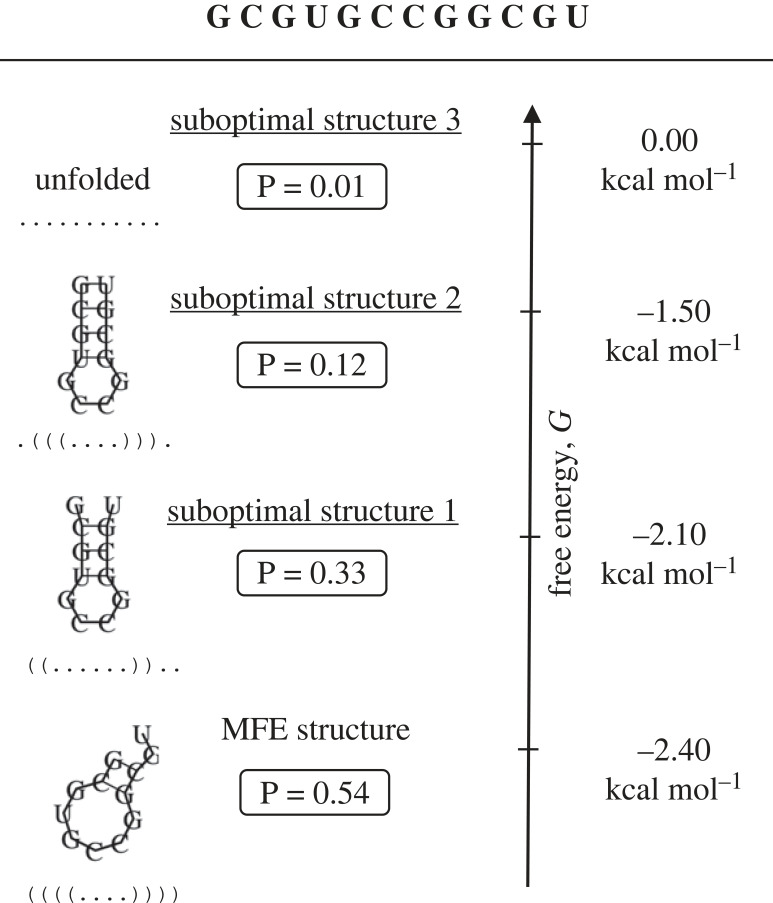
RNA plasticity example. Schematic of an example set of Boltzmann-distributed suboptimal structures for genotype GCGUGCCGGCGU with their corresponding free energies *G* and normalized probabilities *P*. The unfolded case is included as a suboptimal structure with characteristic free energy *G* = 0.0 kcal mol^−1^. Secondary structures and energies are calculated using the ViennaRNA subopt command with default values [7].

In our discussions so far, and in most of the GP map literature more widely, GP maps are deterministic many-to-one mappings, in which each genotype maps to one phenotype. For RNA, the MFE fold represents this deterministic phenotype [1,5,15]. However, in nature, phenotypes can be non-deterministic (ND), either when the environment is variable, or even in a constant environment. For example, RNA folding is stochastic in a constant environment because of the inherent thermal fluctuations of the molecules at finite temperature [15]. Therefore, the nucleotide interactions create a range of possible folds described by the Boltzmann distribution. The structural phenotype of an RNA molecule is in fact ND because it is fluctuating in time through Boltzmann-distributed suboptimal structures [15]. Seen in another way, at any one instant, a large set of RNAs of the same genotype, placed in an environment of temperature *T*, will exhibit a range of folded structures, where the amount of genotypes with fold *p* will be proportional to the Boltzmann probability *P*(*p*|*g*) = exp((*G*_ens_ − *G*_*p*_)/*kT*), where *G*_ens_ is the ensemble free energy (*G*_ens_ = −*kT* · ln*Z* with partition function *Z*) and *G*_*p*_ is the free energy that corresponds to structure *p* [15] (cf. figure 1). Ancel & Fontana’s highly influential work referred to this as RNA plasticity, and studied its GP map [15]. They introduced an intrinsic feature of the plastic RNA GP map called plastogenetic congruence, which is a correlation between a genotype’s suboptimal structures and the MFE structures of its point-mutation neighbours [15]. This feature was also shown to create a negative correlation between RNA plasticity and robustness [15].

Non-determinism is not only found in RNA, but is ubiquitous in biological systems [16]. Macromolecules such as proteins can also assume more than one folded structure [17]. Similarly, on the level of function, it has been shown that a single RNA sequence or a single enzyme can perform multiple functions [18–20]. Beyond the molecular scale, non-determinism also exists: genetically identical microbial cells can have varying phenotypes [21,22], for example due to stochastic gene expression [23]. Across scales, non-determinism could influence evolutionary processes. For example, studies have proposed that it can expedite evolution, but also lead to confinement to less evolvable sequences [15,24–27]. However, there are still many open questions about non-determinism’s impact on evolution [28,29].

The strength of the GP map framework lies in its ability to clearly quantify the sequence-to-structure relationship [5]. The study of these maps has advanced greatly due to the rapid improvement of computational power and tools witnessed over recent decades [30]. But also particularly important, has been the formulation of a general quantifying framework. Quantities such as robustness and evolvability have been key to the exploration of how different deterministic GP maps compare and the finding of deterministic GP map’s quasi-universal properties, which has eventually resulted in significant progress in understanding variation [5,30]. For the ND case, the work from Ancel and Fontana [15] as well as further more recent work [31–36] that includes a ND phenotype in the context of GP maps, has improved our understanding but has not fully addressed how concepts such as robustness, evolvability and neutral spaces can be quantified for the ND case. We define a quantifying framework for non-determinism given by known phenotype probability distributions. In other words, we study the case of non-determinism as stochastic. We expect the ND GP map quantities to be consistent with averages of their deterministic counterparts, and that the framework can be a baseline for further exploration of ND GP maps in general, of any biological context, which could help bring answers on the relationship between non-determinism and evolution.

The terms plasticity [15], promiscuity [30], intra-genotypic variability [37], non-determinism [5] and most recently, ‘probabilistic GP map (PrGP map)’ [35] have been used to describe many-to-many GP maps. Here we will use ‘ND’. The ND GP map we use is defined as a many-to-many mapping where the phenotypes of each genotype form a probability distribution, and for clarity we will refer to a general deterministic GP map, as ‘D GP map’. In the case of RNA, the MFE structure is usually taken to be the deterministic phenotype since it has the highest probability of folding and we will therefore compare our results from the full ND map with this MFE map. The work is structured as follows: first, we mathematically define ND quantities that are designed to correspond to averages of deterministic quantities over an ensemble. Then we validate this correspondence for both RNAs of *L* = 12 (conventional sequence-structure mapping) and *L* = 30 (RNAshapes [38,39]). Following that, the deterministic quantities and their relationships are compared with the ND case to gain a deeper insight into the differences between the RNA ND GP map and the deterministic GP map. The computational methods used to construct the ND GP maps are explained at the end of this paper.

## 2. Results and discussion

In order to generalize GP map measurements such as robustness, evolvability and neutral space size to ND GP maps we take a stochastic approach. The stochastic versions of these measurements will quantify the averages of the corresponding deterministic quantities over the ND ensemble. This definition is general enough to be used in any biological context for which an ND GP map can be constructed. We use short RNA structures as a demonstration and validation of this approach.

### 2.1. Neutral spaces and robustness

In a deterministic GP map, the neutral set of a phenotype is the set of all genotypes that map to it. Its size as a fraction of genotype space is often referred to as the phenotypic frequency [13]. In the ND GP map, we can define neutral sets in terms of the average frequency $\tilde{\;f_{\;p}}$ of a phenotype *p* over the ND probability distribution (as in [35,36,40]):

2.1 $${\tilde{f}}_{\;p}=\frac{1}{K^{L}}\sum_{g\in G_{\;p}}P(p|g),$$

where $G_{\;p}$ is the set of genotypes that contain the phenotype *p* in their ND ensemble and *P*(*p*|*g*) is the probability that genotype *g* gives phenotype *p*. *K*^*L*^ is the total number of genotypes in genotype space, where *K* is the genotype alphabet size and *L* is the genotype sequence length. For RNA, the ensemble is Boltzmann distributed and for sequences of length *L*, the total number of genotypes in genotype space is 4^*L*^ since RNA has an alphabet of 4 nucleotides.

Robustness quantifies the insensitivity of a phenotype to genotypic mutations, and can be defined at the genotypic or phenotypic level [13]. In D GP maps, genotypic robustness *ρ*_*g*_ corresponds to the fraction of genotypic point-mutation neighbours that leave the phenotype unchanged (also referred to as neutral neighbours) [13]

2.2 $$\rho_{g}=\frac{1}{(K-1)L}\sum_{g^{\prime }\in N_{g}}\delta_{g^{\prime }p},$$

where $N_{g}$ is the set of all point-mutation neighbours of *g*, *p* is the phenotype of *g* and *δ*_*g*′*p*_ equals 1 if the genotype *g*′ maps to phenotype *p*, and 0 otherwise. The normalization (*K* − 1)*L* is the total number of point-mutation neighbours in genotype space. In RNA, the number of such neighbours for any genotype is 3*L*. If we want to generalize this definition to the ND case we need to consider the probability that *g* gives rise to phenotype *p* and the probability that genotypes in the mutational neighbourhoods of *g* also give rise to that phenotype. Thus, we define the ND genotypic robustness as

2.3 $${\tilde{\rho }}_{g}=\frac{1}{(K-1)L}\sum_{\;p\in P_{g}}P(p|g)\sum_{g^{\prime }\in N_{g}}P(p|g^{\prime }),$$

where $P_{g}$ is the set of phenotypes *p* in the ND ensemble of genotype *g*, and $\sum_{g^{\prime }\in N_{g}}$ is the sum over all point-mutation neighbours *g*′ of *g*, which form the set $N_{g}$. The maximum of ${\tilde{\rho }}_{g}$ occurs when *g* only maps to one phenotype, and this phenotype is also the only phenotype for *g*′.

The deterministic phenotypic robustness *ρ*_*p*_ is the genotypic robustness (equation (2.2)) averaged over all genotypes that have phenotype *p* [1,13]

2.4 $$\rho_{\;p}=\frac{1}{K^{L}\cdot f_{\;p}}\sum_{g\in G_{\;p}}\rho_{g},$$

where *G*_*p*_ is the neutral set of *p* with size |*G*_*p*_| = *K*^*L*^ · *f*_*p*_. For the ND phenotypic robustness, we formulate an average of the ND robustness of a specific phenotype *p* over the ND neutral set size, resulting in the following definition:

2.5 $${\tilde{\rho }}_{\;p}=\frac{1}{K^{L}\cdot {\tilde{f}}_{\;p}\cdot (K-1)L}\sum_{g\in G_{\;p}}P(p|g)\sum_{g^{\prime }\in N_{g}}P(p|g^{\prime }),$$

where the ND neutral set size is expressed using equation (2.1), as $K^{L}\cdot {\tilde{f}}_{\;p}$. Equation (2.5) is analogous to the ND phenotype robustness definition recently proposed by Sappington & Mohanty [35].

### 2.2. Evolvability

The capacity of a genotype or phenotype to produce phenotypic diversity is quantified by its ‘evolvability’ [13]. For the deterministic case, the genotypic evolvability of a given genotype *g* can be defined as the number of different structures found in its point-mutational neighbourhood [13]. The genotypic evolvability for the genotype *g* of phenotype *p* can therefore be written as

2.6 $$e_{g}=\sum_{\;p^{\prime }\neq p}(1-\Pi_{g^{\prime }\in N_{g}}(1-\delta_{g^{\prime }p^{\prime }})),$$

where, as before, $N_{g}$ is the set of all point-mutation neighbours of *g*, and *δ*_*g*′*p*′_ equals 1 if the genotype *g*′ maps to the phenotype *p*′, and 0 otherwise. For the ND case the *δ*_*g*′*p*′_ is replaced by the probability *P*(*p*′|*g*′), resulting in an average of the number of different phenotypes in the neighbourhood of *g*. The average of each evolvability is weighted according to the probability that this phenotype is initially represented by *g*

2.7 $${\tilde{e}}_{g}=\sum_{\;p\in P_{g}}P(p|g)\sum_{\;p^{\prime }\neq p}(1-\Pi_{g^{\prime }\in N_{g}}(1-P(p^{\prime }|g^{\prime }))),$$

where the sum $\sum_{\;p^{\prime }\neq p}$ is over all possible phenotypes *p*′ in the ND GP map except *p*.

The phenotypic evolvability of a phenotype *p* counts all different phenotypes in the mutational neighbourhood of *p* as a whole, or in other words, of all genotypes with phenotype *p* [13]. For the deterministic case, we can write this as

2.8 $$e_{\;p}=\sum_{\;p^{\prime }\neq p}(1-\Pi_{g\in G_{\;p}}\Pi_{g^{\prime }\in N_{g}}(1-\delta_{g^{\prime }p^{\prime }})).$$

Note the additional product compared with equation (2.6) to make sure we are considering all *g*s with *p*, represented by the neutral set *G*_*p*_. The ND phenotypic evolvability ${\tilde{e}}_{\;p}$ represents the average number of accessible phenotypes from *p*, meaning the set of genotypes in $G_{\;p}$. It can be formulated by replacing the *δ*_*g*′*p*′_ in equation (2.8) with the product of probabilities *P*(*p*′|*g*′)*P*(*p*|*g*) and accounting for all genotypes in $G_{\;p}$

2.9 $${\tilde{e}}_{\;p}=\sum_{\;p^{\prime }\neq p}(1-\Pi_{g\in G_{\;p}}\Pi_{g^{\prime }\in N_{g}}(1-P(p^{\prime }|g^{\prime })P(p|g))).$$

**Figure 2.**
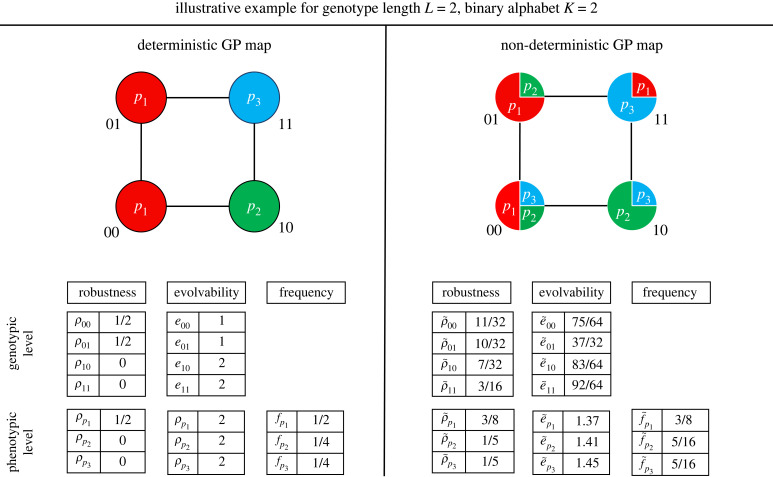
Schematic depiction of simple deterministic and ND GP maps, and associated properties. For an extremely simple genotype space of size 4 (genotype length *L* = 2 and alphabet size *K* = 2) we illustrate the difference between deterministic (D) and non-deterministic (ND) GP maps. The relative area that a phenotype occupies in a genotype node represents the probability that this phenotype arises from this genotype in the ND phenotype ensemble. Each node in the deterministic mapping is, like for the RNA MFE GP map, the most probable phenotype in the ND case. The robustness and evolvability of genotypes and phenotypes, as well as the phenotypic frequency are calculated for both the deterministic and ND case using equations (2.1)–(2.9). A detailed step-by-step calculation of the quantities is shown in the electronic supplementary material, S1.

A simple GP map schematic such as in figure 2 exemplifies the difference between the deterministic and ND case. The schematic shows the deterministic GP map as the map that assigns the most probable phenotype from the ND map for each genotype, which is analogous to how the MFE GP map of RNA relates to its ND GP map.

### 2.3. Validation of non-deterministic quantities

**Figure 3.**
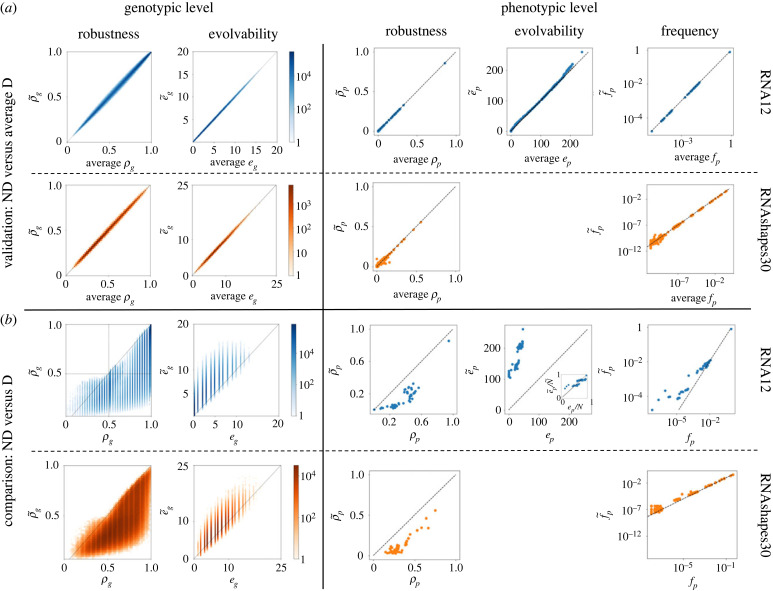
Non-deterministic framework validation and comparison. (*a*) The definitions of non-deterministic robustness $\tilde{\rho }$, evolvability $\tilde{e}$ and phenotypic frequency ${\tilde{\;f}}_{\;p}$ quantities are validated by comparing them with the averages of the equivalent deterministic quantities for RNA12 and RNAshapes30, across 500 samples of the ND GP map in both cases. (*b*) Direct comparison between non-deterministic quantities on the ND GP map and the deterministic quantities on the MFE GP map. The phenotypic evolvability cannot be calculated through sampling methods [41], therefore we do not present evolvability plots for RNAShapes30. The phenotypic evolvability comparison for RNA12 includes an inset for normalized phenotypic evolvability *e*_*p*_/*N*, ${\tilde{e}}_{\;p}/\tilde{N}$, which shows a very close correspondence of these two quantities (see full-sized figure in electronic supplementary material).

We have defined the ND quantities in equations (2.1), (2.3), (2.5), (2.7) and (2.9) to give us the averages of the deterministic quantities over a large number of realizations of the phenotype ensemble. To validate these ND definitions computationally we use short RNA, a biologically realistic computational GP map for molecular evolution. The ND GP maps for RNA of *L* = 12 and RNAshapes of *L* = 30 [39] are computationally constructed using the ViennaRNA package (see Methods). The validation consists of comparisons between the ND quantities for the ND GP map and the deterministic quantities averaged over a large sample (*N* = 500) of GP map realizations: each realization is produced by drawing a sample from the Boltzmann distribution of each genotype, producing a single phenotype for each genotype in a given realization. As can be seen in figure 3*a* the ND definitions of robustness, evolvability and frequency match the averages of the deterministic quantities as expected, both at the genotypic and phenotypic levels. The phenotypic evolvability cannot be calculated with sampling methods as discussed in [41], therefore *e*_*p*_ and ${\tilde{e}}_{\;p}$ are not computed for RNAshapes30.

### 2.4. Comparison of non-deterministic genotype–phenotype map properties with their minimum free energy counterparts

We next compare the ND quantities in the ND GP map with their deterministic MFE counterparts. The latter are the GP map quantities that have been studied extensively in the RNA GP map literature (see Introduction). Figure 3*b* shows these comparisons.

At the genotypic level, figure 3*b* shows similar behaviour for both RNA12 and RNAshapes30. We find that genotypes with high genotypic robustness in the MFE GP map tend to also have high robustness in the ND GP map. However, we find that in most cases the D genotypic robustness is higher than the ND genotypic robustness ($\rho_{g}\geq {\tilde{\rho }}_{g}$), except for a low-density section of genotypes with ${\tilde{\rho }}_{g}>\rho_{g}$ in the regime ${\tilde{\rho }}_{g}$, *ρ*_*g*_ < 0.5. Similarly, genotypes with high genotypic evolvability in the MFE GP map tend to also have high evolvability in the ND GP map. However, genotypic evolvability is higher in the ND case (i.e. ${\tilde{e}}_{g}\geq e_{g}$) for the majority of genotypes. Therefore, we conclude that (most) genotypes in the ND GP maps of RNA secondary structure will on average be less robust and more evolvable than the same genotypes in the corresponding MFE GP map.

At the phenotypic level, figure 3*b* shows each corresponding quantity for the same phenotype. In the RNA12 ND GP map (energy range 15*k*_*B*_*T*, see Methods) there exist a total of $\tilde{N}=271$ phenotypes, while in the deterministic case there are only *N* = 48. Therefore, we can only compare the GP map quantities for the 48 phenotypes present in both maps. Given the result at the genotypic level, it is not surprising that phenotypic robustness is lower in the ND case than in the deterministic one. Phenotypic evolvability, however, shows a sharp increase, meaning that the same phenotypes are surrounded by many more phenotypes. However, this is a function of the increased total number of phenotypes found in the ND GP map, as shown by an inset plot comparing the fractions of all possible phenotypes that are discovered in the neighbourhood of a given phenotype *p*, which we will refer to as the normalized phenotypic evolvabilities $\tilde{e_{\;p}}/\tilde{N}$ (for the ND GP map) and *e*_*p*_/*N* (for the D GP map). These normalized evolvabilities are similar in the MFE and ND GP maps (inset), but phenotypes at the low-evolvability end have slightly higher normalized evolvabilities in the ND case and thus a higher fraction of phenotypes is accessible from them. In all phenotypic plots for RNA12 the outlier at the top right of the distribution represents the unfolded structure, which has a disproportionately large neutral space [14]. The phenotypic frequencies exhibit $\tilde{\;f_{\;p}}>f_{\;p}$ for the most part, particularly for low values of *f*_*p*_ and ${\tilde{\;f}}_{\;p}$. This means that low-frequency phenotypes have larger neutral spaces in the ND GP map than in the MFE GP map, for both RNA12 and RNAshapes30.

### 2.5. Structural properties of the non-deterministic genotype–phenotype map

**Figure 4.**
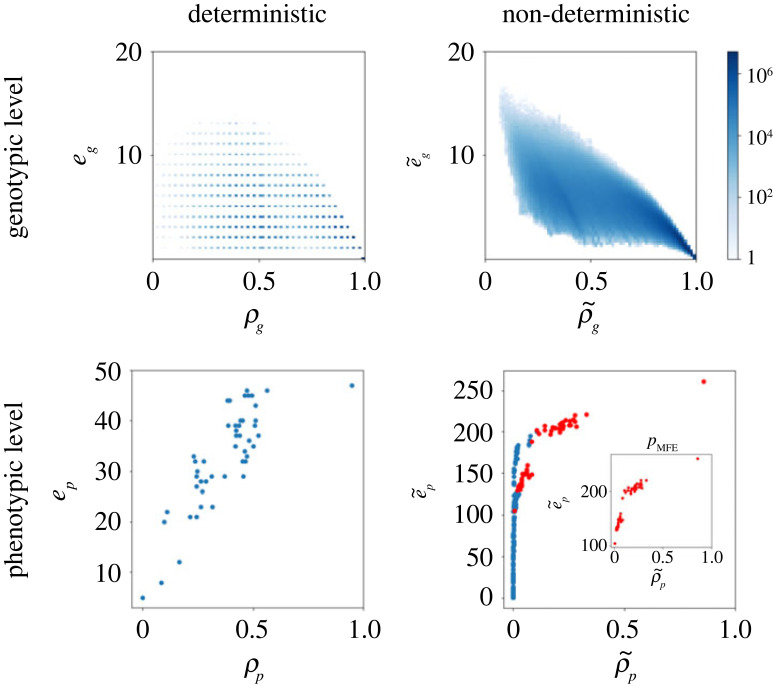
RNA12 robustness versus evolvability. At the genotypic level evolvability and robustness exhibit negative correlations, with a Pearson correlation coefficient of −0.73 in the MFE GP map, and −0.85 in the ND GP map. At the phenotypic level, the correlation between evolvability and robustness is positive with Pearson correlation coefficients 0.81 (MFE GP map) and 0.61 (ND GP map). The structures that appear in both the MFE and ND GP map are highlighted in red in the ND phenotypic evolvability/robustness plot.

Next we determine whether the structural properties that have been observed across several GP maps, including the RNA MFE GP map (see Introduction) also hold in the ND GP map. First, the so-called ‘bias’ observed in many GP maps describes the highly skewed distribution of phenotypic frequencies [5]. In most GP maps, the vast majority of genotypes maps to a small number of phenotypes, and the remainder maps to many different phenotypes. This property is also found in the ND RNA GP maps because the phenotypic frequency values, ${\tilde{f}}_{\;p}$, differ by orders of magnitude, as shown in figure 3 (as found in [35,36,40]). Second, many GP maps, including the RNA MFE GP map, exhibit a negative correlation of genotypic evolvability and robustness and a positive correlation of phenotypic evolvability and robustness [13]. The negative correlation in the genotypic case is expected as the neighbourhood of an individual genotype cannot contain many instances of the same phenotype and also instances of many other phenotypes. A trade-off is therefore inevitable. The positive correlation at the phenotypic level, however, is a consequence of the shape and size of neutral networks, as well as the organization of information into biological sequences [13,42,43]. These relationships also hold in the case of the ND GP map, as can be seen in figure 4 for RNA12. At the genotypic level, the expected MFE GP map negative correlation is present (Pearson correlation coefficient of −0.73) and a similar negative correlation can be observed in the ND GP map (Pearson correlation coefficient of −0.85). At the phenotypic level, the expected positive correlation is observed for the MFE GP map (Pearson correlation coefficient of 0.81). The ND GP map also shows a positive correlation (Pearson correlation coefficient of 0.61), but the points are somewhat differently distributed. If we distinguish MFE phenotypes (highlighted in red, and also plotted separately in the inset) among all phenotypes in the ND GP map, we recover a distribution that mirrors the MFE case more closely. The phenotypes that only appear in the ND GP map form a long tail with very low robustnesses but a wide range of evolvability values.

**Figure 5.**
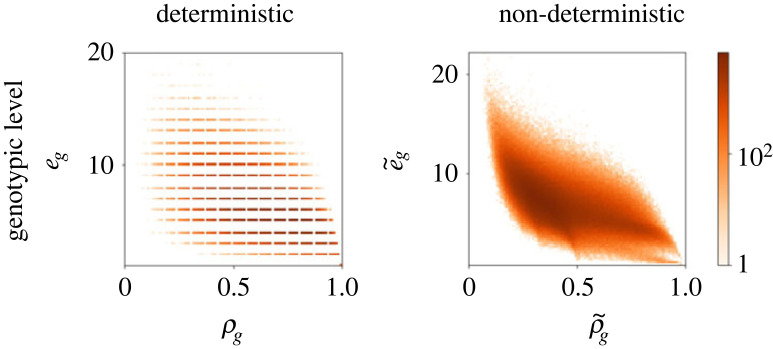
RNAshapes30 robustness versus evolvability. The MFE GP map shows a negative correlation between the genotypic robustness *ρ*_*g*_ and genotypic evolvability *e*_*g*_, with a Pearson coefficient of −0.56. A negative correlation is also observed in the ND GP map with Pearson coefficient of −0.64.

In figure 5, the RNAshapes30 display a similar relationship between evolvability and robustness at the genotypic level, in the form of negative correlations for both the MFE and ND GP maps (Pearson coefficient of −0.56 for the MFE GP map and −0.64 for the ND GP map). Again, because the phenotypic evolvability cannot be calculated with sampling methods [41], we do not study the correlations at the phenotypic level for RNAshapes30. These results indicate that the commonly observed correlations between evolvability and robustness are also present in the ND GP map.

**Figure 6.**
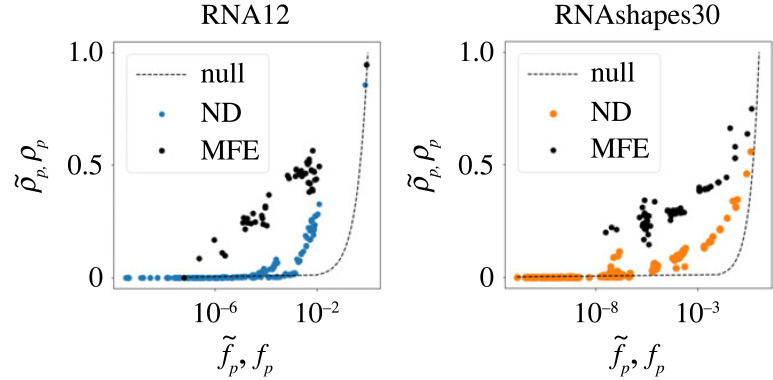
The presence of neutral correlations: phenotypic robustness versus phenotypic frequency for RNA12 and RNAshapes30. Neutral correlations are still present in the ND GP maps for high phenotype frequencies, ${\tilde{\rho }}_{\;p}\propto \log\;{\tilde{f}}_{p}$. The robustnesses of low-frequency phenotypes equal those of the null model, ${\tilde{\rho }}_{\;p}={\tilde{f}}_{\;p}$. In the MFE GP maps, the neutral correlations are present for all frequencies, *ρ*_*p*_ ∝ log *f*_*p*_, as previously observed [14].

Many GP maps, including the RNA MFE GP map, display neutral correlations [14]. One might expect these to also be found in the ND GP map, due to plastogenetic congruence. This concept, introduced by Ancel & Fontana [15] relates mutational neighbourhood of a structure to its Boltzmann distribution (see electronic supplementary material). To verify that the ND GP map also contains neutral correlations we verify whether ${\tilde{\rho }}_{\;p}\propto \log{\tilde{f}}_{\;p}$ holds, as has been found for D GP maps [4,10,14]. Figure 6 shows the relationship between these two quantities, and verifies the presence of correlations, as for most phenotypes the robustness ${\tilde{\rho }}_{\;p}$ is significantly larger than the null model representing a random distribution of phenotypes with ${\tilde{\rho }}_{\;p}={\tilde{f}}_{\;p}$, as previously found for the D and recently also the ND GP maps [14,35]. We also observe, in both the RNA12 and RNAshapes30 ND GP maps, the presence of the recently observed biphasic robustness scaling [35]: for higher-frequency phenotypes, neutral correlations are present ${\tilde{\rho }}_{\;p}\propto \log{\tilde{f}}_{\;p}$ but suppressed compared with the MFE GP map, which follows from the lower phenotypic robustness found in figure 3; in lower-frequency phenotypes, the correlations disappear and the relationship becomes analogue to the null model ${\tilde{\rho }}_{\;p}\propto {\tilde{f}}_{\;p}$ [35].

## 3. Conclusion

The study of GP maps and their structural properties provides an important perspective on evolutionary processes that complements the widely studied concept of the fitness landscape [30,44]. For example, a determinant feature that has driven the evolution of natural functional RNAs is the accessibility of the phenotypes in the GP map which depends on the neutral networks and their distribution [5]. This suggests that the GP map structure and its implications for variation can be just as important as selection pressures [5], as new phenotypes need to be accessible in order to be selected. Robustness, evolvability and neutral spaces have become definitions in the general framework to quantify the relationship between the genotype and phenotype in GP maps, and have been key to the exploration of the quasi-universal structural properties seen in GP maps in different biological contexts [1,45]. Our contribution is a generalization of this framework to the ND GP map, for which each genotype has an ensemble of possible phenotypes that form a probability distribution. In molecular evolution, non-determinism plays a fundamental role since molecules are exposed to thermal fluctuations and produce ND phenotypes in the form of a statistical distribution of folded structures. The study of ND GP maps therefore provides a more realistic picture of variation and its impact on molecular evolution. Such a ND framework can also be applied on the functional level and beyond the molecular scale. For example, there exist experimental observations of enzyme promiscuity [20], or of microbial ND phenotypes [21–23]. The results discussed in this paper provide a foundation for further research on ND GP maps and future work should apply the quantities defined here to the full ND version of other widely studied GP maps, for example proteins or GRNs. A particularly interesting avenue to explore would be how these ND quantities impact evolutionary dynamics on the ND GP maps. In the simplest models, the ND fitness can be computed as the fitness average over the ND phenotype ensemble [28]. The impact of this many-to-many fitness landscape on evolutionary trajectories and how functional structures have evolved is an open question of biological interest. In addition, further work should build on Ancel and Fontana’s work [15] and address the error-threshold problem for a range of structures on the full RNA ND GP map. The ND characteristics we have defined here provide a foundation for such a research agenda.

## 4. Methods

### 4.1. RNA12

The ND GP map is constructed by computing the Boltzmann ensemble of suboptimal structures and their probabilities for each RNA sequence of length *L*. For the case of RNA *L* = 12, the folds are calculated for every sequence through ViennaRNA with all parameters set to default values (e.g. the temperature *T* = 37°C), using the ViennaRNA suboptimal function [7] and the Boltzmann probabilities of these are obtained using the partition function. The energy range for the suboptimals is 15*k*_*B*_*T* as in [46] to be consistent with the RNAshapes data. The final ND GP map is constructed by mapping each genotype sequence to its ensemble of structures in the energy range (including unfolded structure), as well as their respective normalized probabilities.

### 4.2. RNAshapes for length *L* = 30

The RNAshapes calculations rely on the implementation in [46], which uses the ViennaRNA package (v. 2.4.14) [7]. This is faster than the original RNAshapes program for short sequences and thus allows us to work with larger samples of sequences and structures. We use the same parameters as in [46] (shape level 2, temperature *T* = 37°C, energy range 15 *k*_*B*_*T*, sequence length *L* = 30, no isolated base pairs). Unlike [46], however, we compute the deterministic map by taking the most frequent structure in the Boltzmann ensemble as the deterministic folded structure, without additional requirements on its relative frequency.

Since there are 4^30^ ≈ 10^18^ genotypes of sequence length *L* = 30, we have to rely on sampling: we simply work with 10^6^ randomly generated sequences (10^5^ for the average case, which is computationally more expensive due to the necessary repetitions). For the plots showing genotype robustness and evolvability, we simply plot one value for each sequence in the sample—thus, not all possible genotypes are in the sample, but we obtain an overview of common genotypic robustness and evolvability values for a large random sample of genotypes. For the phenotypic robustness estimates, we simply approximate each sum over all sequences by a sum over our sequence sample. This approach was tested for RNA12, where we have exact data as a reference (shown in the electronic supplementary material, section S1.1). To investigate whether the chosen sequence sample is large enough, we analysed how robust our results are to sub-sampling (shown in the electronic supplementary material, section S1.2). Phenotypic evolvabilities are not estimated since they cannot be inferred reliably from samples with current techniques [41].

Phenotypic frequencies were also estimated from a random sequence sample. However, phenotypic frequency calculations are faster than robustness calculations because they do not require us to examine the mutational neighbours of each sequence, and so we used a larger sample of 10^8^ sequences. Note that our reliance on sampling methods means that quantities for low-frequency phenotypes, which do not appear in the sample, cannot be estimated.

## Data Availability

Access code and data from the GitHub repository: https://github.com/paulagrcg/The-non-deterministic-genotype-phenotype-map-of-RNA-secondary-structure.
